# Autoimmune liver disease - are there spectra that we do not know?

**DOI:** 10.1186/1476-5926-10-9

**Published:** 2011-09-12

**Authors:** Hind I Fallatah, Hisham O Akbar

**Affiliations:** 1 King Abdul Aziz University Hospital, Jeddah, Saudi Arabia

**Keywords:** Autoimmune liver disease, autoimmune hepatitis, primary biliary cirrhosis, primary sclerosing cholangitis, autoimmune cholangitis, cholestasis, hepatocellular, ursodeoxycholic acid

## Abstract

Autoimmune liver diseases (AILDs) are common leading causes for liver cirrhosis and terminal stage of liver disease. They have variable prevalence among patients with liver disease and have two major clinical and biochemical presentations. Autoimmune hepatitis (AIH) is the typical example of hepatocellular AILD, but it can also be presented under a cholestatic pattern. AIH has a scoring diagnostic system and respond in most cases to the treatment with prednisolone and azathioprine. Primary biliary cirrhosis (PBC) is the second most common AILD, with a cholestatic presentation and characterized by positive antimitochondrial antibody (AMA). It has an excellent response and long term outcome with the administration of ursodeoxycholic acid (UDCA). Another AILD that is thought to be a variant of PBC is the autoimmune cholangitis, being a disease that has biochemical and histological features similar to PBC; but the AMA is negative. Primary sclerosing cholangitis (PSC) is a rare entity of AILD that has a cholestatic presentation and respond poorly to the treatment, with the ultimate progression to advance liver cirrhosis in most patients. Other forms of AILD include the overlap syndromes (OS), which are diseases with mixed immunological and histological patterns of two AILD; the most commonly recognized one is AIH-PBC overlap (AIH-PSC overlap is less common). The treatment of OS involves the trial of UDCA and different immunosuppressants. Here we present three case reports of unusual forms of chronic liver diseases that most likely represent AILD. The first two patients had a cholestatic picture, whereas the third one had a hepatocellular picture at presentation. We discussed their biochemical, immunological and histological features as well as their response to treatment and their outcomes. Then, we compared them with other forms of AILD.

## Background

Autoimmune liver diseases (AILD) are a group of immunologically induced hepatic damage that are either hepatocellular or cholestatic [1,2]. The hepatocellular forms are characterized by a significant elevation of the serum alanine aminotransferase (ALT) and aspartate aminotransferase (AST), as compared with the biliary enzymes, together with elevated serum bilirubin. The cholestatic forms involve either the intra- or the extra-hepatic biliary systems or both. Cholestasis will ultimately cause impairment of bile formation and/or bile flow which may clinically present with fatigue, pruritus, and jaundice [1,2]. The biochemical markers include increases in serum alkaline phosphatase (ALP) and gamma-glutamyl transpeptidase (GGT), followed by conjugated hyperbilirubinemia, at more advanced stages. Cholestasis is considered chronic if it lasts more than 6 months [3]. Most chronic cholestatic diseases are purely intra-hepatic [3,4]. They are considered as different disease entities based on the clinical, laboratory and histological features [3,4]. In many instances, however, some of the histological and or the pathological features of one AILD disease may follow another; in addition, the two disease entities may coexist in the same patient [3,4]. Those forms of presentations are defined as overlap syndromes (OS) [3,4]. The presence of the overlap patterns of cholestatic liver disease suggests that those diseases may represent spectra of a common or similar immunological and pathological process that causes the hepatobiliary damage [1,5].

Autoimmune hepatitis (AIH) is a chronic relapsing remitting necroinflammatory disease associated with elevation of the serum immunoglobulins and autoantidobies [2,6]. The disease mostly affects children and young adults, but can also affect older people [7-9]. AIH has various clinical presentations from asymptomatic disease to advance liver cirrhosis or severe forms of acute liver failure [6-9]. The usual biochemical presentation of AIH is a hepatocellular pattern (more prominent elevation of the serum ALT and AST as compared to serum ALP and GGT), but in many cases AIH can present with a cholestatic picture that may confuse AIH with other autoimmune cholestatic liver diseases [6,9-12]. The diagnosis of AIH is based on the scoring system that was established and modified by the International Autoimmune Hepatitis Group [13,14]. Simplified diagnostic scoring criteria have been suggested [15]. The treatment of choice for AIH is corticosteroids and azathioprine. The majority of treated patients with AIH will achieve remission with this therapy; in some reports, 65% and 80% at 18 month and 3 years, respectively [2,16,17]. In the remaining 20% - standard therapy unresponsive AIH - other form of immunosuppressant medication have been tried, like mycophenolate mofetil, and cyclosporine, and found to be effective in some patients [2,16].

Primary biliary cirrhosis (PBC) is a non-suppurative destructive granulomatous cholangitis characterized by involvement of the small intra-hepatic bile ducts [2,4,18]. PBC mostly affect middle-aged females. Many patients with PBC are asymptomatic whereas others may complain of fatigue and pruritus. The liver biochemical parameters will show cholestatic abnormality of the hepatic enzymes. The serum immunoglobulin profile will show elevated serum IgM [18,19]. Positive serum antimitochondrial antibodies (AMA) are the characteristic hallmark for PBC it is found in 90-95% of patients [2-4,18]. In the diagnosis of PBC, liver biopsy is not mandatory in the presence of cholestatic pattern of liver enzymes and positive serum AMA; but it may help in staging the disease [3,18]. The treatment of choice for patients with PBC is ursodeoxycholic acid (UDCA). It has been found in several studies that UDCA, at a dose of 13-15 mg/kg/day, is effective in improving the liver biochemistry, and delay the histological progression of the disease. It was also found to be effective in the improvement of survival and reduce the need for liver transplantation [2,3,18]. UDCA is not effective for relieving the pruritus in PBC -- other agents, like cholestyramine and rifampicin, are used for the purpose. Several other medications (including cyclosporine, corticosteroids, azathioprine, thalidomide, mycophenolate mofetil, chlorambucil, penicillamine, methotrexate, and colchicine) have been tried in patients who had inadequate response to UDCA, but none of them showed promising effects [2,3,18], apart from budesonide combined with UDCA in an early stage of the disease [20].

Autoimmune cholangitis (AIC) - or antimitochondrial antibody-negative primary biliary cirrhosis (AMA negative PBC) - is an autoimmune cholestatic liver disease that was described in 1987. Over the following years, an increasing number of patients with similar presentations have been observed [4]. AIC has distinctive features from PBC in that the AMA is negative, the serum IgM is normal, whereas showing a higher frequency of positive in antinuclear antibodies (ANA) and smooth muscle antibodies (SMA) [4,21]. The subsequent identification of more cases of AIC that mimicked PBC raised the possibility that AIC may be a transitional stage of PBC [22,23]. In some patients who had PBC, the detection of AMA may be a false negative if lower sensitivity assays are used and those patients will be misdiagnosed as AIC [24]. Earlier reports on the treatment of AIC had shown poor response to the treatment both to corticosteroids and UDCA [23]. However, in a recent study, it was shown that AIC patients had a similar response rate to that of patients with AMA plus PBC [25].

Primary sclerosing cholangitis (PSC) is a chronic, progressive cholestatic liver disease of unknown etiology, characterized by an inflammatory and fibrotic structuring process affecting both intra- and extra-hepatic bile ducts. It is a disease that is more common in men at their 40s, with a male:female ratio of 2:1 [2,3,26]. In 80% of patients PSC is associated with inflammatory bowel disease, more commonly with ulcerative colitis. The diagnosis of PSC is made in the presence of a cholestatic biochemical profile and the typical cholangiographic findings of multifocal strictures and segmental dilatations, and secondary bile ducts changes on magnetic resonance cholangiography (MRCP), endoscopicretrograde cholangiography or percutaneous transhepatic cholangiography. The causes of secondary sclerosing cholangitis have to be excluded [4,26]. Elevated serum IgG and positive autoantibodies are other features for PSC. The most frequently encountered autoantibody in PSC is the antineutrophil cytoplasmic antibodies (ANCA), in 26-94% of PSC patients. Although not specific, the liver biopsy finding may help to support the diagnostic [4,26]. Patients who had biochemical, immunological and histological features for PSC, but normal cholangiographic examination, are classified as small duct PSC [26,27]. Although less promising in PSC compared to PBC, UDCA is the only medication to date found to be effective in PSC patients. It was shown that UDCA results in biochemical improvement [28]. Histological improvement of PSC on the treatment with UDCA has been demonstrated too [29]. In PSC patients who had dominant structure with severe biochemical deterioration, or recurrent septic cholangitis, percutaneous or endoscopic cholangiographic approaches can be used to relieve the obstruction [4,26].

The autoimmune overlap syndromes (AOS) are supposed to arise as distinctive cholestatic liver diseases or an outcome of two coexisting AILDs [4]. AOS account for 13.9-18% of all patients with AILDs [30,31]. The pathogenesis of the AOS is not clear [32]. AIH-PBC overlap is the most common form [32]. They are thought to arise from AIH and PBC developing simultaneously or one preceding the other [33]. Diagnostic scoring criteria for AIH-PBC have been developed [34] and recently a simplified diagnostic score have been suggested [35].

AIH-PSC overlap is a disorder with ill-defined immune mediated backgrounds [3]. It is more common in children and adolescent [3,32]. Although no specific diagnostic criteria have been established for AIH-PSC overlap, in the largest reported number of patients with this syndrome -- they had clinical, biochemical and immunological features of AIH coexisting with radiological evidence of PSC [36]. The treatments of AOS are empiric and involve the use of both immune suppressive therapy and UDCA [3,30,32,37]. Patients with AIH-PBC overlap have treatment response and prognostic outcome that is poorer compared with those with isolated AIH and BPC; but patients with AIH-PSC overlap have treatment response and prognosis that have worse prognosis when compared to patients only with AIH and otherwise better when compared to patients only with PSC [37,38].

## Case presentations

### First patient

A 27-year-old Chadian lady, mother for 2 children, had a history of progressive jaundice and itching for 2 years. She denied the ingestion of medication and herbal medicines, previous similar attacks, jaundice during pregnancy, contact with jaundice patient and blood transfusion. She also denied a family history of liver disease or similar presentations. On physical examination, she was slim (weight: 36 kg) with stable vital signs. Examination was only positive for deep jaundice and scratch marks all over the body; the rest of the examination was unremarkable. The lab tests showed normal CBC apart from mild anemia; hemoglobin of 11.3 g/dl, white blood cells (WBC) 5.9 k/μl and platelets (Plat) of 190 k/μl. The prothrombin time (PT) of 15 seconds was normal (11-14).

The liver function tests showed ALT 40 U/L (normal 30-65), AST 74 U/L (normal 15-37), ALP 231 U/L (normal 50-136), GGT 321 U/L (normal 5-85), total protein 60 g/L (normal 64-82), albumin 25 g/L (normal 35-50), and total and direct bilirubin 325 μmol/L (normal 0-17) and 274 μmol/L (0-5), respectively.

She had negative immunological profile for ANA done by indirect immunofluorescence (IIF), SMA done by ELISA, ANCA done by IIF, liver kidney microsomal-1 (LKM-1) done by ELISA, as well as negative serology for hepatitis B virus (HBSAg, HBeAg, HBeAb, HBcAb), hepatitis C virus (HCVAb) and HIV. The serum immunoglobulin-G (IgG) level was 23 (normal 5.4-16.1). The serum copper, ceruloplasmin, 24 hour urine copper, serum iron and transferrin saturation were all normal. Ultrasound abdomen and MRCP were normal. Liver biopsy showed evidence of interphase hepatitis stage 3/6, with focal intrabiliary steatosis and mild intra cellular cholestasis. The histological activity index was 5/18.

**Figure 1:**
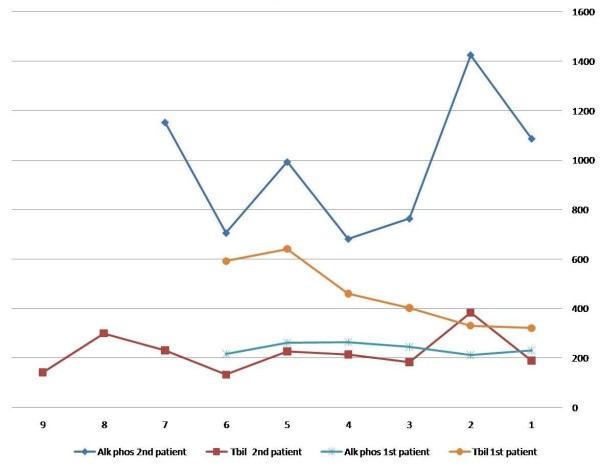
**Results of the serum alkaline phosphatase (Alk phos) and bilirubin levels (T Bil) for the first two patients during the follow-up**.

She started treatment with prednisolone (60 mg daily) and UDCA (250 daily); nevertheless, for over 6 month she did not show any improvement of the symptoms or liver enzymes profile (maintaining normal to 1.5 times normal ALT and AST) but continued to have progressive cholestasis (Figure 1). Over the next 6 months of follow up, the symptomatology worsed. She developed moderate ascites that progressed to diuretic refractory ascites over a few months, recurrent bacterial peritonitis and 4 attacks of stage III-IV hepatic encephalopathy. Prednisolone was tapered down, and then stopped; finally, she was selected for liver transplantation, however she died while in the waiting list.

### Second patient

The second patient was a 30-year-old male, a Saudi security officer, who presented a history of progressive jaundice for 2 years. He had unremarkable past history, denying drug or alcohol abuse, and medications, including herbal medicines. There was no family history of liver disease or history of contact with jaundiced patients. His physical examination showed normal vital signs. He had deep jaundice, but the rest of the general examination was normal. The chest, the cardiovascular, and the abdominal examinations were normal. His baseline workup showed CBC (WBC 8.4 k/μl, Hg11.5 g/l, Plat 373), LFT (AST 531 U/L, ALT 250 U/L, ALP 682 U/L, GGT 205 U/L, TBil 344, Direct Bil 278, albumin 17, total protein 80), PT 13.3, and the renal functions were normal. The ultrasound examination of the abdomen showed hepatomegaly, but there were no evidence of biliary obstruction.

The ANA, SMA, AMA, LKM-1, HBV serology, HCV serology and the HIV testing were all negative. The serum IgG level was 25. Testing for Wilson's disease, by serum copper, ceruloplasmin and 24 hours urine copper, revealed normal results. Similarly, the serum iron and the total iron binding capacity (TIBC) and the transferrin saturation were normal. He had MRCP that showed a normal biliary system cholangiography. A liver biopsy was performed and it detected marked sinusoidal dilatation, infiltration of the biliary tracts with chronic inflammatory cells (mostly lymphocytes and some plasma cells), associated with bile duct damage. There was also chronic inflammatory cell infiltration of the hepatic lobules. The hepatocytes showed cholestasis. In some areas some of the hepatocytes were replaced by macrophages. There was also a mild portal and sinusoidal fibrosis.

He was given a trail of prednisolone (40 mg, daily) and UDCA (250 mg, three times a day), but excessive acne and skin rash appeared. Prednisolone was reduced to 30 mg and azathioprine (50 mg, daily) was started then gradually increased (to100 mg, daily). The treatment was maintained for more than 8 months; however, he had only transient improvement in the liver enzymes and bilirubin levels in the first few weeks of the treatment; nonetheless, latter on he lost that response while still on prednisolone and azathioprine. The serum ALT and AST were maintained at the 3-4 times above the normal, but the ALP and bilirubun progressively increased (Figure 1); so prednisolone and azathioprine were discontinued. Because of severe symptomatic cholestasis, he was selected for liver transplantation.

### Third patient

The third patient was a 36-year-old Indian male who had progressive jaundice and itching for 10 month. He also noticed darkening of the urine and he also complained of intermittent melena, alternating with fresh rectal bleeding, over the past few months. Six month later, he had right upper quadrant abdominal pain of moderate severity. Two month prior to his clinical appointment, he start having progressive abdominal distention, and lower limb edema, for which he was given diuretics in a polyclinic; the ascites had improved. He did not have history of fever or hepatic encephalopathy during that period. There was no history of medication or herbs intake, drug or alcohol abuse, contact with jaundiced patients and family history of liver disease. His general examination was remarkable for jaundice, palmar erythema, spider nevi, itching marks and mild lower limb edema. The chest examination revealed right-sided pleural effusion. The cardiovascular examination depicted a short systolic murmur. On the abdominal examination, he had a moderate amount of ascites and splenomegaly. The lab data showed: CBC (WBC 3.82 k/μl, Hg12.7 g/dl, Plat 106 k/μl), PT 17.9 seconds, LFT(AST 223 U/L, ALT 74 U/L, ALP 174 U/L, GGT 215 U/L, TBil 144 μmol/L, DBil 12 μmol/L, albumin 22 g/L, TP 66 g/L), the renal functions were normal. The immunological profile was negative for ANA, LKM-1, AMA, ANCA, HBV, HCV and HIV. The SMA was weakly positive. The serum IgG was elevated 26.6 g/L and the serum IgM was normal. Tests for Wilson's disease, by serum and urine copper studies, and by ceruloplasmin testing, were normal. Similarly, the serum iron, transferrin, TIBC and transferrin saturation were also normal. The level of alpha-1 antitrypsin was also normal. The ultrasound examination of the abdomen showed hepatosplenomegaly and moderate amount of ascites. The echocardiogram was normal. Upper gastrointestinal endoscopy showed grad III esophageal varices. Endoscopic examination of the colon revealed internal piles, but the colonic mucosa was normal. Because of the clinical and laboratory evidence of advanced cirrhosis, the liver biopsy was deferred. He was thought to have late stage of AIH and was given a trial of prednisolone (30 mg, daily). Over the following 3 month of treatment, he had neither clinical nor biochemical improvements. His liver function tests at the end of the 3 month of the treatment with prednisolone showed ALT 247 U/L, AST 181 U/L, ALP 174 U/L GGT 167 U/L and total Bil 98 μmol/L. He was advised to undergo liver transplantation; therefore, he traveled back to India to have it done there.

## Discussion

The above three patients had atypical forms of chronic liver disease that lead to decompensated advanced cirrhosis in two of them. The immunological profile and the serum antibodies testing were performed for all of them in different medical centers, on at least two occasions, and the same above result was obtained. The first patient was a young female who started to have jaundice at the age of 25 years. With the negative viral profiles and the negative workup for metabolic diseases she was thought to have an atypical presentation of AIH, because of the cholestatic liver enzyme profile and negative autoantibodies. However, the elevated serum IgG of 1.43 times the upper normal and the liver biopsy features supported the possibility of AIH. In the most recent simplified criteria for the diagnosis of AIH, serum IgG of 1.1 times the normal is accepted in the diagnosis of AIH and a level of 1.44 the normal was found to be the best diagnostic predictor for AIH [15]. AIH with negative autoantibodies is not unusual [13,39]. The treatment response of this form of AIH compared to autoantibodies positive AIH have not been previously addressed. AIH usually respond partially, or even completely, to the treatment with steroids and azathioprine [2,16]. The absence of response to prednisolone in this patient even after increasing of the dose to 2 mg/kg sounds against AIH. Because of the cholestatic presentation AIC (AMA negative PBC) was another possibility; but, once again, in the previously reported cases of AIC autoantibodies were part of the diagnostic features. In addition, AIC have been found to respond to the treatment with steroids, azathioprine and UDCA [23,25]. This was not the case in this patient. On the other hand, the rapid progression to cirrhosis in relatively short time in spite of the treatment with UDCA sounds against AIC. PSC was almost ruled out by negative cholangiography and absent histological feature for PSC.

The second patient was a young male who had a similar presentation to the first patient. Because of elevated serum IgG and liver biopsy features, AIH was also considered the most likely diagnosis. AIH is more common in females but it is a disease that have been frequently reported in males as well [7,9,10]. The diagnosis of AIH was further supported by the transient partial response to prednisolone and azathioprine in the first few weeks of the treatment. AIC (AMA negative PBC) was another possibility that was considered. It has been shown in the previous reports on AIC that it is less responsive to the treatment as compared to AIH [23,40]. Being a male with atypical histological features and absence of response to UDCA make AIC unlikely. Similar to the first patient, PSC was ruled out because of absent cholangiographic and histological features which could support it. Because he had intractable symptoms with severe cholestasis he was selected to liver transplantation [3,40].

The third patient had hepatocellular elevation of the liver enzymes. This, together with high serum IgG level and weakly positive SMA, raises the possibility of AIH in this patient. The liver biopsy was not performed because of the advance stage of the disease. Upon his presentation this patient had already evidence of advanced de-compensated cirrhosis. This may be the reason for his poor response to the treatment. In the previous reports on AIH patients with de-compensated cirrhosis although they have less chance of response to the treatment as compared to compensated patients they can still have complete or near complete response with favorable outcome [7,9]. Because of the hepatocellular presentation, PBC, AIC and PSC were not likely to be the diagnosis in this patient.

AOS of autoimmune liver disease were unlikely to be the diagnosis in the three patients, because of the absent typical immunological and biochemical features of both types of AOS. Some of the non-autoimmune chronic liver diseases have been reported to be associated with elevated serum immunoglobulins and variable levels of positive autoantibodies [41,42]. Drug induced liver disease or toxic hepatitis can cause both cholestatic or hepatocellular hepatic abnormalities [43,44], but these have been ruled out by the detailed frequent questioning of the three patients. Another issue regarding toxic hepatitis is that most injures are of acute forms, and only few medications (like miodarone and methotrexate) have been reported to cause liver fibrosis and cirrhosis [45,46]. Familial forms of intra-hepatic inherited cholestatic syndromes were unlikely in the first and the second patient, because of the age of presentation, and because both of them had negative family history of liver disease [3]. Non-alcoholic fatty liver disease was not a possibility because of the young age of the three patients, short time or progression to cirrhosis and presence of cholestatic picture in the first two patients sounds against cryptogenic cirrhosis [47]. On the other hand, cryptogenic cirrhosis was reported to be associated with diabetes mellitus, hyperlipidemia and high body mass index, which was not the case in all the three patients [47].

## Conclusions

In many instances autoimmune liver diseases have been thought to represent spectra or variable presentation of similar disease entity [3]. Although the immunological features in the three patients we studied were not so strong, the possibility that they had a form or forms of autoimmune liver disease that is not responding to the usual immunosuppressants and that progress rapidly to advanced cirrhosis at relatively young age is there. Future reporting of similar cases and trails of immune suppressants other than prednisolone and azathioprine in such patients may help to identify an effective treatment of such patients avoiding them the need of liver transplantation.

## Consent

Written informed consent was obtained from for the publication of these Case Reports. Consent was directly made by the patients in the cases of the second and third patients. Respecting the first patient, the consent was obtained from her sister, with the family agreement. Copies of the written consent documents are available for review by the Editor-in-Chief of this journal, and they may be requested to the authors at any time.

## Competing interests

The authors declare that they have no competing interests.

## Authors' contributions

Both HIF and HOA participated in the clinical follow up, in the management of the three patients, in the literature review, and in the writing and editing of the manuscript. All authors read and approved the final content of the manuscript.

## Authors' information

HIF: Dr Hisham O Akbar MBCh B, FRCPCanada, Associate Professor, Consultant Gastroenterologist and Hepatologist, Director of the Gastroenterology and Hepatology Section, King Abdul Aziz University Hospital, Jeddah, Saudi Arabia, member of the Saudi Gastroenterology Association, member of the Saudi Association for Internal Medicine, member of the APASL.

HOA: Dr Hind I Fallatah, MBCh B, Arab Board and Saudi Board of Internal Medicine, MACP. Consultant Gastroenterologist and Hepatologist, King Abdul Aziz University Hospital, Jeddah Saudi Arabia, member of the Saudi Gastroenterology Association, member of the Saudi Association for Internal medicine, Member of the APASL.
